# Use of leading practices in US hospital antimicrobial stewardship programs

**DOI:** 10.1017/ice.2022.241

**Published:** 2023-06

**Authors:** Edward A. Stenehjem, Barbara I. Braun, Salome O. Chitavi, David Y. Hyun, Stephen P. Schmaltz, Mohamad G. Fakih, Melinda M. Neuhauser, Lisa E. Davidson, Marc J. Meyer, Pranita D. Tamma, Elizabeth S. Dodds-Ashley, David W. Baker

**Affiliations:** 1 Division of Infectious Diseases and Epidemiology, Intermountain Healthcare, Salt Lake City, Utah; 2 Division of Healthcare Quality Evaluation, The Joint Commission, Oakbrook Terrace, Illinois; 3 The Pew Charitable Trust, Washington, DC; 4 Ascension Healthcare, St. Louis, Missouri; 5 Division of Healthcare Quality Promotion, National Center for Emerging and Zoonotic Infectious Diseases, Centers for Disease Control and Prevention, Atlanta, Georgia; 6 Division of Infectious Diseases, Department of Medicine, Atrium Health, Charlotte, North Carolina; 7 Infection Prevention and Clinical Pharmacy, Southwest Health System, Cortez, Colorado; 8 Department of Pediatrics, Johns Hopkins University School of Medicine, Baltimore, Maryland; 9 Division of Infectious Diseases and International Health, Duke University Medical Center, Durham, North Carolina

## Abstract

**Objective:**

To determine the proportion of hospitals that implemented 6 leading practices in their antimicrobial stewardship programs (ASPs). Design: Cross-sectional observational survey.

**Setting:**

Acute-care hospitals.

**Participants:**

ASP leaders.

**Methods:**

Advance letters and electronic questionnaires were initiated February 2020. Primary outcomes were percentage of hospitals that (1) implemented facility-specific treatment guidelines (FSTG); (2) performed interactive prospective audit and feedback (PAF) either face-to-face or by telephone; (3) optimized diagnostic testing; (4) measured antibiotic utilization; (5) measured C. difficile infection (CDI); and (6) measured adherence to FSTGs.

**Results:**

Of 948 hospitals invited, 288 (30.4%) completed the questionnaire. Among them, 82 (28.5%) had <99 beds, 162 (56.3%) had 100–399 beds, and 44 (15.2%) had ≥400+ beds. Also, 230 (79.9%) were healthcare system members. Moreover, 161 hospitals (54.8%) reported implementing FSTGs; 214 (72.4%) performed interactive PAF; 105 (34.9%) implemented procedures to optimize diagnostic testing; 235 (79.8%) measured antibiotic utilization; 258 (88.2%) measured CDI; and 110 (37.1%) measured FSTG adherence. Small hospitals performed less interactive PAF (61.0%; P = .0018). Small and nonsystem hospitals were less likely to optimize diagnostic testing: 25.2% (P = .030) and 21.0% (P = .0077), respectively. Small hospitals were less likely to measure antibiotic utilization (67.8%; P = .0010) and CDI (80.3%; P = .0038). Nonsystem hospitals were less likely to implement FSTGs (34.3%; P < .001).

**Conclusions:**

Significant variation exists in the adoption of ASP leading practices. A minority of hospitals have taken action to optimize diagnostic testing and measure adherence to FSTGs. Additional efforts are needed to expand adoption of leading practices across all acute-care hospitals with the greatest need in smaller hospitals.

Antimicrobial stewardship programs (ASPs) are critical infrastructure to improve antibiotic prescribing in hospitals. They are designed to optimize clinical outcomes while minimizing unintended consequences of antibiotic use, including adverse drug events, *Clostridioides difficile* infections (CDI), and emerging antibiotic resistance.^1^

In 2014, the Centers for Disease Control and Prevention (CDC) called on all US hospitals to implement ASPs and released the *Core Elements of Hospital Antibiotic Stewardship Programs* (Core Elements) to guide hospitals in achieving this goal.^2^ The Core Elements describe structural and process components associated with successful ASPs. In 2015, the US National Action Plan for Combating Antibiotic Resistant Bacteria (CARB) set a goal to implement the Core Elements in all hospitals that receive federal funding.^3^ The CDC updated its Core Elements in 2019 to emphasize the importance of hospital leadership, commitment, accountability, pharmacy expertise, actions such as prospective audit and feedback (PAF), local guidelines for common conditions, and antibiotic use tracking using the National Healthcare Safety Network (NHSN) Antimicrobial Use option.^4^

To support the National Action Plan for CARB, The Joint Commission established ASP standards for its accredited hospitals effective January 2017.^5^ In 2017, the Agency for Healthcare Research and Quality (AHRQ) Safety Program for Improving Antibiotic Use began a pragmatic quality-improvement program that produced free, setting-specific, tool kits for ASPs.^6,7^ The Centers for Medicare and Medicaid Services (CMS) added federal regulations for hospital antibiotic stewardship programs to the conditions of participation in 2019.^8^

These combined efforts appear to have been successful in establishing ASPs in hospitals; self-reported data from NHSN annual hospital surveys revealed that 91% of acute-care hospitals had all 7 Core Elements in place in 2020, compared to only 41% in 2014.^9^ Although most hospitals have a basic infrastructure, it is important to ensure that ASPs are implementing effective approaches that strengthen and advance their existing programs.

To identify promising, evidence-based leading ASP practices, The Joint Commission and The Pew Charitable Trusts convened an in-person meeting of experts and key stakeholder organizations in May 2018.^10^ Leading practices can be described as best and emerging interventions that complement, strengthen, or go beyond traditional interventions conducted by ASPs. The group identified 6 leading practices (3 established or emerging practices and 3 measurement-related practices) that top-performing ASPs should be performing to improve care for patients: (1) development and implementation of facility-specific treatment guidelines (FSTGs), (2) interactive prospective audit and feedback (also known as handshake stewardship), (3) optimizing diagnostic testing (also known as diagnostic stewardship), (4) measurement of antimicrobial use using days of therapy per 1,000 days present or patient days, (5) measurement of hospital-onset CDI, and (6) measurement of adherence to FSTGs.

In this study, we assessed the proportion of Joint Commission–accredited hospitals that have implemented these 6 leading practices of antimicrobial stewardship, and we identified hospital characteristics associated with these practices.

## Methods

This cross-sectional observational study was guided by 9 expert advisors who (1) helped develop the web-based questionnaire, (2) established minimum necessary requirements to determine whether a hospital has implemented a leading practice, and (3) advised on data interpretation. Table 1 presents the leading practices operational descriptions.

**Table 1.** Short Names and Operational Definitions of Leading Practices

Short Name	Operational Definition of What Constituted Meeting the Leading Practice as Defined by the Expert Panel
Facility-specific treatment guidelines	Hospital has developed facility-specific treatment guidelines for community-acquired pneumonia (CAP), urinary tract infection (UTI), skin and soft-tissue infection (SSTI), and sepsis. The hospitals have at least implemented the CAP, UTI, and SSTI guidelines using any of the following formats: e-mail, web site, treatment algorithms (eg, order sets) or pathways built into the electronic health records (EHR), educational conferences, meetings (eg, in-services), face-to-face education (eg, during handshake stewardship rounds), pocket cards, hand-outs, and/or flyers.
Interactive prospective audit and feedback	Hospital performs prospective audit and feedback, whereby a member of the antimicrobial stewardship program (ASP) team provides feedback either face-to-face (handshake stewardship), or by telephone (calling and speaking with the clinician or leaving voice message) or both.
Diagnostic testing optimization	Hospital has implemented procedures to optimize diagnostic testing for *Clostridioides difficile* (*C. difficile*) and urinary tract infections. Also known as diagnostic stewardship.
Antibiotic use measure	Hospital routinely measures days of therapy (DOT) per 1,000 days present or 1,000 patient days.
Hospital-onset *C. difficile* infection measure	Hospital measures hospital-onset *C. difficile* infection rates (CDI).
Facility-specific treatment guideline adherence measure	Hospital measures adherence to facility-specific treatment guidelines for at least 1 of the following: CAP, UTI, SSTI, or sepsis.

### Questionnaire development

We reviewed published literature and previous questionnaires, and we held several advisory panel meetings to establish questionnaire domains and review draft questions.^11^ The draft questionnaire was pilot tested at 22 hospitals in fall 2019 (Supplementary Methods 1 online). To calculate prevalence of leading practices, algorithms linked specific combinations of questionnaire items (Supplementary Methods 2 online).

### Surveyed hospitals

General medical–surgical acute-care hospitals, children’s hospitals, and critical-access hospitals (CAHs) that received accreditation following a full Joint Commission accreditation survey visit in 2018 were eligible for inclusion. Hospitals due for a survey visit in 2019 or 2020 were excluded to reinforce that the study was unrelated to accreditation. The Joint Commission, a not-for-profit organization, accredits ∼3,239 (64.3%) of 5,038 US nonspecialty hospitals: 2,328 (76.9%) of 3,416 general medical-surgical acute-care hospitals, 94 (81.0%) of 116 children’s hospitals, 152 (89.9%) of 169 federally owned hospitals, and 365 (27.3%) of 1,337 of CAHs.^12,13^

Following a hardcopy advance letter to hospitals in January 2020, a 50-item questionnaire was sent by e-mail to the designated ASP leader (Supplementary Methods 3 online). The desired minimum sample size, calculated based on 5% precision and confidence intervals (CIs) of 95% after applying a finite population correction factor, was determined to be 274 hospitals.

### Data analysis

We used R version 3.5 software (R Foundation for Statistical Computing, Vienna, Austria) for data analysis. Sampling weights were used to adjust the results for nonresponse and were applied to the calculation of prevalence for the leading practices. Logistic regression was used to estimate the probability that a sampled hospital had completed the survey as a function of bed-size category (ie, small, ≤100 beds; medium, 100–399 beds; and large, ≥400 beds), location (urban or rural), health-system status (membership in a hospital system or not), and teaching status (major, minor, or nonteaching). The inverse of the predicted probability of response was used as the weight. The mean scores for each practice, both overall and stratified by hospital characteristics, were calculated using these sampling weights. Sampling weights were not applied to frequencies of other descriptive survey findings. *P* values < .05 were considered significant. We used the χ^2^ test to examine differences in response rates by hospital characteristics, and we have provided 95% CIs for the mean scores, overall, and by characteristic.

This project was reviewed by Ethical and Independent Review Services and was determined to be exempt from institutional review board (IRB) review.

## Results

Approximately 1,600 hospitals underwent a full accreditation survey in 2018. Of these, 601 were specialty hospitals and 44 did not have valid contact information. E-mail invitations were sent to 948 eligible hospitals in 48 states. Overall, 288 (30.4%) of 948 hospitals completed the questionnaire, meeting the sample size needed for estimated precision. Respondents came from 47 states.

Among responding hospitals, 82 (28.5%) were small, 162 (56.3%) were medium sized, and 44 (15.3%) were large. Also, 228 (79.2%) hospitals were in urban settings; 26 (9%) were major teaching hospitals; 230 (79.9%) belonged to a healthcare system; 25 were CAHs; and 5 were children’s hospitals. Small hospitals (*P* = .005) and nonteaching hospitals (*P* = .01) were less likely to respond compared to large, teaching hospitals. Healthcare system membership and location were similar between respondents and nonrespondents. (Table 2). Furthermore, 141 respondents (49.0%) reported their role or title as specialists in antimicrobial stewardship or infectious disease (eg, ASP pharmacist, ID clinical pharmacist, or ASP medical director); 125 (43.4%) reported their role or title as nonspecialist pharmacy directors or clinical pharmacists; and 22 (7.6%) reported another role (eg, infection preventionist or director of quality).

**Table 2.** Characteristics of Hospitals that Responded

Hospital Characteristics /Respondents	Respondents (n=288), No. (%)	Nonrespondents (n=660), No. (%)	Total (n=948)	*P* Value
**Belongs to healthcare system** ^**a**^				
No	58 (20.1)	130 (19.7)	188	.96
Yes	230 (79.9)	530 (80.3)	760	
**Hospital size**				
Large, ≥400 beds	44 (15.3)	80 (12.1)	124	.005
Medium, 100–399 beds	162 (56.3)	318 (48.2)	480	
Small, 0–99 beds	82 (28.5)	262 (39.7)	344	
**Teaching status** ^**b**^				
Major	26 (9.0)	38 (5.8)	64	.01
Minor	150 (52.1)	304 (46.1)	454	
Nonteaching	112 (38.9)	318 (48.2)	430	
**Hospital location** ^**c**^				
Urban	228 (79.2)	484 (73.3)	712	.06
Rural	60 (20.8)	176 (26.7)	236	

Note. ASP, antimicrobial stewardship program; ID, infectious diseases.

Percentages are unweighted. The χ^2^ test was used to test for the significance of differences in hospital characteristics.

a System indicates whether a hospital is affiliated with a healthcare system. A multihospital health care system is 2 or more hospitals owned, leased, sponsored, or contract managed by a central organization (AHA data dictionary 2018).

b Teaching hospitals are those with Council of Teaching Hospitals designation (COTH). Minor teaching hospitals are those approved to participate in residency and/or internship training by the Accreditation Council for Graduate Medical Education (ACGME), or American Osteopathic Association (AOA) or those with medical school affiliation reported to the American Medical Association. Nonteaching hospitals are those without COTH, ACGME, AOA or medical school (AMA) affiliation (AHA data dictionary 2018).

c Hospital location indicates rural or urban location based on Metropolitan Statistical Area (MSA) designation. A rural location is defined as located outside an MSA, as designated by the US Office of Management and Budget (OMB). An urban area is a geographically defined, integrated social and economic unit with a large population nucleus (AHA data dictionary 2018).

### Prevalence of leading practices

Weighted estimates of the prevalence of the leading practices are provided in Table 3 with stratification by hospital characteristics. Implementation across all 6 leading practices was as follows: Only 3 hospitals (1%) indicated that they had implemented no practices. However, 16 hospitals (5.6%) indicated that they had implemented 1 practice; 37 hospitals (12.9%) indicated that they had implemented 2 practices; and 69 hospitals (24.0%) indicated that they had implemented 3 practices. Furthermore, 68 hospitals (23.6%) indicated that they had implemented 4 practices; 56 hospitals (19.4%) indicated that they had implemented 5 practices; and 39 hospitals (13.5%) indicated that they had implemented 6 practices. The median number of leading practices implemented across hospitals was 4 (interquartile range, 3–5).

**Table 3.** Prevalence of Leading Practices and Associated Hospital Characteristics

Hospital Characteristic	No.	Implementing Facility-Specific Treatment Guidelines^b^	Performing Interactive Prospective Audit and Feedback	Optimizing Diagnostic Testing for CDI and UTI	Measuring Antibiotic Use^b^	Measuring Hospital-onset CDI	Measuring Adherence to ≥ 1 Treatment Guideline^a^
		% (95% CI)	% (95% CI)	% (95% CI)	% (95% CI)	% (95% CI)	% (95% CI)
**Size**							
Large	44	58.2 (49.7–66.8)	88.3 (82.8–93.9)	44.5 (35.9–53.1)	93.1 (88.7–97.5)	97.6 (95.0–100)	38.0 (29.6–46.5)
Medium	162	58.0 (53.7–62.4)	76.7 (72.1–80.4)	39.7 (35.4–44.0)	85.3 (82.2–88.4)	91.7 (89.3–94.2)	40.3 (36.0–44.5)
Small	82	49.3 (44.2–54.4)	61.0 (56.0–66.0)	25.2 (20.7–29.6)	67.8 (63.0–72.5)	80.3 (76.2–84.3)	32.5 (27.7–37.3)
*P* value		.41	.002	.030	.001	.004	0.49
**Location**							
Urban	228	57.0 (53.4–60.6)	79.4 (76.4–82.3)	38.1 (34.5–41.6)	83.6 (80.9–86.3)	90.8 (88.7–92.9)	40.3 (36.8–43.9)
Rural	60	48.6 (42.5–54.7)	52.6 (46.5–58.6)	25.8 (20.5–31.2)	69.1 (63.5–74.8)	81.0 (76.2–85.8)	28.0 (22.6–33.5)
*P* value		.25	<.001	.065	.033	.085	.072
**Teaching status**							
Major	26	62.0 (50.5–73.4)	92.5 (86.3–98.7)	54.2 (42.4–65.9)	92.4 (86.2–98.7)	100.0 (100–100)	46.0 (34.3–57.8)
Minor	150	58.7 (54.3–63.2)	73.5 (69.5–77.6)	36.4 (32.0–40.8)	83.5 (80.2–86.9)	91.6 (89.1–94.2)	40.3 (35.8–44.7)
Nonteaching	112	49.8 (45.2–54.3)	68.2 (63.9–72.4)	30.6 (26.4–34.6)	74.2 (70.2–78.2)	83.1 (79.7–86.6)	32.6 (28.4–36.9)
*P* value		.29	.008	.11	.033	.31	.31
**Belongs to system**							
No	58	34.3 (27.8–40.8)	73.2 (67.1–79.2)	21.0 (15.5–26.6)	69.9 (63.6–76.2)	86.3 (81.6–91.0)	37.8 (31.2–44.4)
Yes	230	60.2 (56.8–63.6)	72.1 (69.0–75.3)	38.6 (35.2–42.0)	82.4 (79.7–85.1)	88.7 (86.5–90.9)	36.9 (33.5–40.2)
*P* value		<.001	.87	.008	.078	.65	.90
**Overall**	288	54.8 (51.7–57.9)	72.4 (69.6–75.2)	34.9 (31.9–37.9)	79.8 (77.3–82.3)	88.2 (86.2–90.2)	37.1 (34.1–40.1)

Note. CAP, community-acquired pneumonia; UTI, urinary tract infection; SSTI, skin and soft-tissue infection; CDI, *Clostridioides difficile* infection.

a Facility-specific treatment guidelines included CAP, UTI, SSTI, and sepsis.

b Measured as days of therapy per 1,000 days present or 1,000 patient days.

### Facility-specific treatment guidelines

Overall, 268 hospitals (93.1%) developed FSTGs for at least 1 inpatient condition. The most frequently addressed conditions were community-acquired pneumonia (CAP) (n = 246 hospitals, 85.4%), sepsis (n = 232 hospitals, 80.6%), urinary tract infection (UTI) (n = 215 hospitals, 74.7%), and skin and soft-tissue infection (SSTI) (n = 199 hospitals, 69.1%). Furthermore, 161 hospitals (55.9%) developed FSTGs for CAP, UTI, SSTI, and sepsis (Supplementary Table 1 online). Hospitals not in a health system were least likely to have met the criteria for this leading practice (34.3%; 95% CI, 27.8%–40.8%; *P* < .001) (Table 3). Guidelines were generally implemented by treatment algorithms or pathways built into the electronic health records (EHR) system via order sets.

### Interactive prospective audit and feedback

Overall, 239 hospitals (83.0%) reported having any process for prospective audit and feedback (PAF). Approaches used to provide frontline staff with feedback varied widely. Recommendations were commonly provided by the ASP pharmacist (n = 198, 68.8%) using some combination of telephone (n = 224, 77.8%), face-to-face (n = 198, 68.8%), text message (n = 155, 53.8%), or EHR alert (n = 104, 36.1%). Most hospitals (n = 198, 68.8%) reviewed orders for all units; 142 (49.3%) reviewed orders for all antimicrobials, and 123 (42.7%) reviewed orders 4–5 days per week (Table 4).

**Table 4.** Approaches for Prospective Audit and Feedback

Approaches for Prospective Audit and Feedback	No. (%) (n=288)
Hospital performs prospective audit and feedback	239 (83.0)
**Healthcare professional(s) who usually provided prospective audit and feedback** ^**a**^	
ASP pharmacist	198 (68.8)
ASP physician	61 (21.2)
ASP pharmacist and ASP physician together	52 (18.1)
Other, pharmacist or physician	96 (33.3)
Nurse or nurse practitioner	7 (2.4)
Hospitalist	11 (3.8)
Other	28 (9.7)
**Method(s) of communication for prospective audit and feedback** ^**a**^	
E-mail	29 (10.1)
Telephone (calling and speaking with the clinician or leaving voice message)	224 (77.8)
Text message or instant message	155 (53.8)
EHR alerts or notes	104 (36.1)
Face-to-face (handshake stewardship)	198 (68.8)
Other	9 (3.1)
**Does ASP team review antibiotic orders for all units of the hospital or specific units**	
Reviews orders for all units or locations	198 (68.8)
Reviews orders for specific units or locations	37 (12.8)
Unknown	3 (1.0)
Missing	1 (0.3)
**In units where prospective audit and feedback is performed, does ASP team review orders for all antimicrobials or just specific drugs/drug classes (eg, carbapenems)**	
ASP reviews orders for all antimicrobials	142 (49.3)
ASP reviews antimicrobial orders for specific drugs or drug classes	94 (32.6)
Unknown	1 (0.3)
Missing	2 (0.7)
**In units where prospective audit and feedback is performed, days per week antimicrobial orders are reviewed by the ASP team**	
1–3 days per week	55 (19.1)
4–5 days per week	123 (42.7)
6–7 days per week	59 (20.5)
Missing	2 (0.7)

Note. ASP, antimicrobial stewardship program; EHR, electronic health record.

Percentages are unweighted.

a Respondents were asked to select all applicable responses.

Regarding the leading practice criteria, 214 hospitals (72.4%) performed interactive PAF whereby an ASP team member provided feedback either by telephone (speaking with the clinician or leaving voice message), face to face, or both. Small hospitals (61.0%; 95% CI, 56.0%–66.0%; *P* = .0018), rural hospitals (52.6%; 95% CI, 46.5%–58.6%; *P* < .001), and nonteaching hospitals (68.2%; 95% CI, 63.9%–72.4%; *P* = .0076) were less likely to have implemented interactive PAF (Table 3).

### Diagnostic testing optimization

Overall, 207 hospitals (71.9%) had procedures in place to optimize the appropriate use of diagnostic tests. Regarding the leading practice criteria, only 105 hospitals (34.9%) had implemented procedures to optimize testing for both *C. difficile* and UTIs (Table 3). Small hospitals (25.2%; 95% CI, 20.7%–29.6%; *P* = .030) and nonsystem hospitals (21.0%; 95% CI, 15.5%–26.6%; *P* = .0077) were less likely to meet this leading practice.

The main strategies used to optimize diagnostic testing for *C. difficile* were laboratory-initiated interventions (n = 165 hospitals, 57.3%) or clinician education sessions (n = 162, 56.3%). Allowing reflex urine cultures only when specific parameters were met (n = 91, 31.6%) and clinician education (n = 87 hospitals, 30.2%) were strategies commonly used to optimize urine-specimen testing. Hospitals frequently (n = 120, 41.7%) used a clinical decision support system to optimize diagnostic testing for CDI though fewer (n = 34, 11.8%) did so for urine-specimen testing (Supplementary Table 2 online).

### Measurement-related practices

Regarding antimicrobial use, 235 (79.8%) hospitals routinely measured days of therapy (DOT) per 1,000 days present or 1,000 patient days. Small hospitals (67.8%; 95% CI, 63.0%–72.5%; *P* = .0010), rural hospitals (69.1%; 95% CI 63.5%–74.8%; *P* = .033), and nonteaching hospitals (74.2%; 95% CI, 70.2%–78.2%; *P* = .033) were less likely to measure antibiotic DOTs (Table 3).

The overall proportion of hospitals measuring hospital-onset CDI (HO-CDI) was high (n = 258, 88.2%). Small hospitals were least likely (80.3%; 95% CI, 76.2%–84.3%; *P* = .0038) to measure HO-CDI. The proportion of hospitals monitoring provider adherence to at least 1 FSTG (ie, CAP, UTI, SSTI or sepsis) was low. Only 110 hospitals (37.1%) met this leading practice, with no differences by hospital characteristics (Table 3). Approximately one-fourth assessed adherence to either UTI (n = 73 hospitals, 25.3%), sepsis (n = 71 hospitals, 24.7%), or CAP (n = 70 hospitals, 24.3%); however, only 46 hospitals (16.0%) assessed adherence to FSTG for SSTI (Supplementary Table 1 online). Some hospitals (n = 59, 20.5%) collected adherence information manually, and 48 hospitals (16.7%) collected information electronically. Adherence results were disseminated to clinicians in formal meetings such as a pharmacy and therapeutics committee or medical staff (n = 109 hospitals, 37.8%), followed by informal approaches such as PAF (n = 63 hospitals, 21.9%), individually in person (n = 57 hospitals, 19.8%), in-service educational lectures (eg, grand rounds; n = 44 hospitals, 15.3%), and using e-mail distribution (n = 29 hospitals, 10.1%).

## Discussion

In this study, we sought to determine what proportion of Joint Commission–accredited hospitals had implemented the 6 leading practices of antimicrobial stewardship previously identified by an expert group.^10^ Overall, these results show encouraging signs that US hospitals are adopting the leading practices of antimicrobial stewardship.

Most hospitals had implemented 1 or more facility-specific guidelines and slightly more than half have guidelines in place for CAP, UTI, SSTI, and sepsis. Similarly, in most hospitals, ASP team members were performing interactive prospective audit and feedback. Interactive prospective audits are powerful interventions to modify clinician practice and optimize treatment. The most common modes of interaction were by phone call, face-to-face, and text messaging. There was, however, considerable variation across hospitals in how often this was done, how many units were included, and which drugs were reviewed. In a similar study in Colorado hospitals, 55% of respondents were performing handshake stewardship.^14^

Most hospitals measured antibiotic use with the recommended days of therapy metric.^15,16^ Enrollment in the CDC NHSN Antimicrobial Use option allows hospitals to electronically capture and submit these data, in partnership with a vendor, and to calculate a standardized antimicrobial administration ratio.^17^ Similarly, most hospitals were measuring CDI rates, likely due to the mandatory NHSN measure in the CMS hospital Inpatient Quality Reporting program.^18^

However, 2 leading practices remain greatly underutilized. The first is measuring adherence to at least 1 FSTG, which was done by only approximately one-third of hospitals. Without data on adherence to treatment guidelines, improvement will be difficult for many hospitals. There are several possible reasons for the underutilization. Often hospitals lack the technical support resources or EHR capabilities to electronically capture adherence data. If these resources are not available, time-consuming retrospective manual data collection is required. Also, no standardized metrics or guidance for measuring FSTG adherence is available. In cases in which nonadherence is identified, it may be difficult to attribute nonadherence to individual prescribers for targeted interventions to change provider behavior. Changing behavior is more difficult to implement than technical changes to electronic systems. Greater understanding of barriers to assessing adherence to local guidelines and readily available tools are critical to improving this practice.

Finally, only one-third of hospitals reported efforts to optimize diagnostic testing for *C. difficile* and UTIs and the subsequent prescribing of unnecessary antibiotics that results from inappropriate testing. This was the lowest overall percentage among the leading practices. This may be because optimizing diagnostic testing requires a multidisciplinary effort that involves adjusting infection control and/or microbiology laboratory protocols. Optimizing testing for *C. difficile* was slightly higher than for urine specimens, likely because CDI rates are publicly reported.^18^ Clinician education as an intervention to improve diagnostic testing was higher for *C. difficile* than for collecting urine specimens. This difference may be because there is more clarity on when and who to test for *C. difficile* and less clarity on when to obtain a urine specimen. This finding is consistent with an infection preventionist survey that found hospitals frequently rejected formed stool submitted for CDI testing but that the use of urine culture stewardship was much lower.^19^

### Variation by hospital characteristics

In this study, the implementation of 4 leading practices was less common among small hospitals. The first 2 practices were interactive prospective audit and feedback and diagnostic stewardship, which may reflect more dedicated roles and established expertise in antimicrobial stewardship at larger hospitals. Although hospitals with fewer providers sometimes have a more collaborative environment, they also have fewer ASP staff with ID training.^20^ In-person feedback may be more challenging in small hospitals where the physician is only present a small portion of the day. Small hospitals may also have less information technology (IT) surveillance capability to target review, although strategies to overcome these limitations have been recommended.^21–24^ Two other practices less common in small hospitals were measurement of CDI and antibiotic use. This may be because CAHs are not yet required to participate in the CMS IQR program and are less likely to enroll in the NHSN Antimicrobial Use option.^18^

Hospitals belonging to a health system more frequently performed 2 leading practices. The first was developing guidelines for 4 conditions and implementing guidelines for CAP, UTI, and SSTI. Health systems can provide centralized resources including ASP clinical expertise for FSTG development as well as the technical staff needed to incorporate FSTGs into EHRs.^25–27^ Similarly, belonging to a system was associated with optimizing diagnostic testing for *C. difficile* and UTIs. Diagnostic testing guidelines can often be integrated into EHR order sets at the system level.

As described, our findings indicated that most hospitals have implemented some, but not all, of the leading practices. Oversight organizations and national public health agencies have played a pivotal role in working to establish prioritized requirements for ASPs, driving demonstrable improvement over time, maintaining antibiotic stewardship in the national spotlight, and modifying prioritized requirements with new data. Now may be the right time for oversight organizations to direct increased attention to ASPs and to help reprioritize resources. Several studies have reported that ASP activities decreased when resources shifted to the COVID-19 pandemic response.^28–32^

Our findings underscore the importance of substantive time and financial commitment from clinical and administration leadership for ASPs at both the health-system and local-hospital levels. Such support can create an infrastructure that will facilitate the dissemination and implementation of best practices and build the personnel and technical capacity for ASPs to achieve local goals, assess guideline adherence, and provide interactive prospective audit and feedback, much of which is carried out by pharmacists. When possible, health-system leaders should centralize these capacities and expertise to provide specialized support for smaller hospitals, for example, through antibiotic stewardship telehealth programs.^21,33^

ASP leaders must tailor the implementation of practices or interventions to the local facility environment and their challenges. ASP leaders should determine that the internal environment would be receptive to the change.^34–36^ ASP leaders can also take advantage of free resources such as the AHRQ tool kits and the CDC antimicrobial stewardship program assessment tool.^7,37^

This study had several limitations. The sample included only hospitals accredited by The Joint Commission. Despite efforts to clarify that this project was unrelated to accreditation, the possibility of a positive response bias exists. A follow-up qualitative study of challenges and facilitators related to implementing these practices in a subsample of respondents will elucidate areas in which the questionnaire was unclear. The overall response rate was likely affected by the COVID-19 pandemic. To adjust for lower response rate in small hospitals, we weighted the analysis of leading practice prevalence. Nonresponding hospitals may have been less advanced in their ASP practices. Another limitation is the potential positive response bias associated with self-reported data. We did not collect information on staffing composition of ASP teams, which could confound interpretations related to hospital characteristics. Although the target respondent was the ASP leader, in some cases infection preventionists may have been more familiar with CDI diagnostic stewardship practices and NHSN-related issues because infection preventionists report these data. Finally, we did not address the ASP’s role in outpatient departments. Hospital ASPs often devote considerable resources to these areas. For example, ASP interventions for outpatient respiratory infections may be more salient for smaller hospitals than certain leading practices such as CDI reporting.

Overall, our findings indicate that many hospital ASPs have implemented effective practices such as facility-specific treatment guidelines for common conditions, engaging in interactive prospective audit and feedback, and measuring antibiotic use and CDI. However, advancing diagnostic stewardship activities and assessing compliance with local guidelines will require additional commitment, resources, guidance, and oversight from internal and external partners to maximize the overall impact of ASPs, especially in smaller hospitals.

## Supplementary material

For supplementary material accompanying this paper visit https://doi.org/10.1017/ice.2022.241. click here to view supplementary material
