# Supplementary material for: Use of leading practices in US hospital antimicrobial stewardship programs
Source: Infect Control Hosp Epidemiol. 2022 Oct 13;44(6):861–8. doi: 10.1017/ice.2022.241 (PMC10262155; doi:10.1017/ice.2022.241)
Supplement: Supplementary file 1 [file S0899823X22002410sup001.docx]

**Online Supplement to MS ICHE-43257 “Use of Leading Practices in United States Hospital Antimicrobial Stewardship Programs 8/18/22**

**eMethods 1-Questionnaire**

**eMethods 2-Algorithms**

**eMethods 3- Questionnaire implementation**

**eTable 1 - Monitoring Adherence for Facility-specific Treatment Guidelines**

**eTable 2 - Mechanisms to Optimize Diagnostic Testing**

**Evaluating the Prevalence of Leading Practices in Antimicrobial Stewardship in Joint Commission-accredited Hospitals in the United States**

**eMethods 1-Questionnaire**

**Final Questionnaire - 01/28/2020**

## Role/ Title of person completing questionnaire

Key abbreviations

ASP = Antimicrobial stewardship program

ASP team = The ASP team refers to the individuals that typically perform the day-to-day antimicrobial stewardship activities and interventions. Most often this refers to a pharmacist(s) and/or physician(s). CDI = *Clostridioides difficile* infection (formerly *Clostridium difficile*)

EHR = Electronic health record

## FACILITY-SPECIFIC TREATMENT GUIDELINES: DEVELOPMENT, IMPLEMENTATION AND FEEDBACK

1. Has your hospital or health system developed facility-specific treatment guidelines for specific inpatient infectious disease conditions? *Select one*

*Note: Facility-specific treatment guidelines refer to clinical practice guidelines or protocols for infectious diseases and / or syndromes that are facility-specific. The guidelines may be written in the form of clinical pathways / algorithms or electronic order sets that address antimicrobial use. These guidelines are often based off national guidelines and are tailored to your local susceptibility report (if possible) and common conditions encountered in the facility. The guidelines often also address diagnostic approaches, such as when to send diagnostic samples and what tests to perform.*

[ ] No, we have not developed facility-specific treatment guidelines. *Skip to question 20*

[ ] Unknown *Skip to question 20*

[ ] Yes

1. Which of the following conditions are addressed in the facility-specific treatment guidelines? *Check all that apply*

[ ] Community-acquired pneumonia

[ ] Hospital or ventilator-associated pneumonia [ ] Skin and soft tissue infection

[ ] Urinary tract infection and / or asymptomatic bacteriuria [ ] Sepsis

[ ] *Clostridioides difficile* infection [ ] Surgical prophylaxis

[ ] Intra-abdominal infections (e.g., appendicitis) [ ] Meningitis

[ ] Febrile neutropenia

[ ] Other, *specify*

1. If you have facility-specific treatment guidelines for community-acquired pneumonia, in what format(s) was the guideline distributed? *Check all that apply*

[ ] Not applicable, do not have facility-specific treatment guidelines for community-acquired pneumonia

[ ] Email

[ ] Web site

[ ] Treatment algorithms (e.g., order sets) or pathways built into the EHR [ ] Educational conferences, meetings (e.g., in-services)

[ ] Face-to-face education (e.g., during handshake stewardship rounds) [ ] Pocket cards, hand-outs, flyers, etc.

[ ] Other, *specify:*

1. If you have facility-specific treatment guidelines for urinary tract infection and / or asymptomatic bacteriuria, in what format(s) was the guideline distributed? *Check all that apply*

[ ] Not applicable, do not have facility-specific treatment guidelines for urinary tract infection and / or asymptomatic bacteriuria

[ ] Email

[ ] Web site

[ ] Treatment algorithms (e.g., order sets) or pathways built into the EHR [ ] Educational conferences, meetings (e.g., in-services)

[ ] Face-to-face education (e.g., during handshake stewardship rounds) [ ] Pocket cards, hand-outs, flyers, etc.

[ ] Other, *specify:*

1. If you have facility-specific treatment guidelines for skin and soft tissue infection, in what format(s) was the guideline distributed? *Check all that apply*

[ ] Not applicable, do not have facility-specific treatment guidelines for skin and soft tissue infection

[ ] Email

[ ] Web site

[ ] Treatment algorithms (e.g., order sets) or pathways built into the EHR [ ] Educational conferences, meetings (e.g., in-services)

[ ] Face-to-face education (e.g., during handshake stewardship rounds) [ ] Pocket cards, hand-outs, flyers, etc.

[ ] Other, *specify:*

1. Has your ASP team formally assessed provider compliance with any of your inpatient-focused facility- specific treatment guidelines in the past 5 years?

*Note: This formal assessment of compliance does not include individual assessment during routine prospective audit and feedback*

[ ] No *Skip to question 20*

[ ] Unknown *Skip to question 20*

[ ] Yes

1. To which of the following facility-specific treatment guidelines does your ASP team monitor adherence? *Check all that apply*

[ ] Community-acquired pneumonia

[ ] Hospital or ventilator-associated pneumonia [ ] Skin and soft tissue infection

[ ] Urinary tract infection and / or asymptomatic bacteriuria [ ] Sepsis

[ ] *Clostridioides difficile* infection [ ] Surgical prophylaxis

[ ] Intra-abdominal infections (e.g., appendicitis) [ ] Meningitis

[ ] Febrile neutropenia

1. How often does your ASP team monitor adherence to facility-specific treatment guidelines for

community-acquired pneumonia?

[ ] Quarterly [ ] Annually

[ ] As needed

[ ] Do not know

[ ] Other, *describe*

1. How often does your ASP team monitor adherence to facility-specific treatment guidelines for

hospital or ventilator-associated pneumonia [ ] Quarterly

[ ] Annually

[ ] As needed [ ] Don’t know

[ ] Other, *describe*

1. How often does your ASP team monitor adherence to facility-specific treatment guidelines for skin and soft tissue infection?

[ ] Quarterly [ ] Annually

[ ] As needed [ ] Don’t know

[ ] Other, *describe*

1. How often does your ASP team monitor adherence to facility-specific treatment guidelines for urinary tract infection and / or asymptomatic bacteriuria?

[ ] Quarterly [ ] Annually

[ ] As needed [ ] Don’t know

[ ] Other, *describe*

1. How often does your ASP team monitor adherence to facility-specific treatment guidelines for sepsis? [ ] Quarterly

[ ] Annually

[ ] As needed [ ] Don’t know

[ ] Other, *describe*

1. How often does your ASP team monitor adherence to *Clostridioides difficile* infection guideline? [ ] Quarterly

[ ] Annually

[ ] As needed [ ] Don’t know

[ ] Other, *describe*

1. How often does your ASP team monitor adherence to surgical prophylaxis guideline? [ ] Quarterly

[ ] Annually

[ ] As needed [ ] Don’t know

[ ] Other, *describe*

1. How often does your ASP team monitor adherence to intra-abdominal infections (e.g., appendicitis) guideline

[ ] Quarterly [ ] Annually

[ ] As needed [ ] Don’t know

[ ] Other, *describe*

1. How often does your ASP team monitor adherence to meningitis? [ ] Quarterly

[ ] Annually

[ ] As needed [ ] Don’t know

[ ] Other, *describe*

1. How often does your ASP team monitor adherence to febrile neutropenia? [ ] Quarterly

[ ] Annually

[ ] As needed [ ] Don’t know

[ ] Other, *describe*

1. How does your ASP team most commonly collect the information used to monitor adherence to facility-specific treatment guidelines?

[ ] Manually - by manually reviewing identified cases (includes medication use evaluations)

[ ] Electronically – by utilizing the EHR to electronically capture adherence data

[ ] Other, *describe*

1. If your ASP team formally assesses guideline adherence, how does your team share the results with clinicians? *Select all that apply*

[ ] Informally during prospective audit and feedback

[ ] In-service or other educational lectures (e.g., grand rounds) [ ] Individually, in-person

[ ] At formal meetings such as Pharmacy and Therapeutics Committee, medical staff meetings,

ASP meetings, department meetings, etc [ ] Email distribution

[ ] Other, *describe*

[ ] We do not share the results

## ENGAGING CLINICIANS WHILE THE PATIENT IS ON THE UNIT

**Pre-authorization**

1. Does your hospital require approval or pre-authorization from the ASP team or infectious disease consultant for specific antibiotics?

*Note: If the prescriber wants a specific antibiotic that requires pre-authorization, the order needs to be approved by the ASP team or infectious disease consultant, not just meet specific clinical criteria.*

[ ] No - none *Skip to question 22* [ ] Unknown *Skip to question 22* [ ] Yes

1. Please check which antibiotics require approval? *Select all that apply*

[ ] Vancomycin [ ] Daptomycin [ ] Linezolid

[ ] Dalbavancin [ ] Oritavancin

[ ] Ceftolozane and tazobactam [ ] Ceftazidime / avibactam

[ ] Colitistin

[ ] Meropenem / Imipenem [ ] Ertapenem

[ ] Levofloxacin / Ciprofloxacin / Moxifloxacin [ ] Piperacillin / tazobactam

[ ] Cefepime

[ ] Ceftazadime [ ] Ceftaroline

[ ] Tigecycline

[ ] Others, *specify*

## Prospective audit and feedback

1. Does your ASP team or hospital perform prospective audit and feedback in any capacity? *Select one*

*Note: Prospective audit and feedback, also known as post-prescription review or concurrent review, is a process for reviewing the need and choice of antimicrobial agents for individual patients after they have been prescribed while the patient is in the hospital. Prospective audit and feedback is different*

*from an antibiotic “time out” because the audits are conducted by staff trained in antimicrobial stewardship trained and not the treatment team. The process for prospective audit and feedback is often complex and is modified to meet the needs of the hospital.*

[ ] No, we do not perform prospective audit and feedback *Skip to question 31*

[ ] Unknown *Skip to question 31*

[ ] Yes, we have a process for prospective audit and feedback

1. When performing prospective audit and feedback, does your ASP team review antibiotic orders for all units of the hospital or specific units? *Select one*

[ ] We review antimicrobial orders for all units or locations [ ] Unknown

[ ] We review antimicrobial orders for specific units or locations. *List excluded units*

1. In those units where prospective audit and feedback is performed, how many days per week are the antimicrobial orders reviewed by the ASP team? *Select one*

[ ] 1-3 days per week [ ] 4-5 days per week [ ] 6-7 days per week

1. Relative to when the antimicrobial was prescribed, at what point in time are the antimicrobial orders typically reviewed on weekdays? *Select one*

[ ] As soon as possible after the antimicrobial was prescribed [ ] 48-72 hours after the antimicrobial was prescribed

[ ] More than 72 hours after the antimicrobial was prescribed

[ ] Reviewed at multiple time points (e.g., 24 and 72 hours). *Please explain*

[ ] Other, *describe*

1. In units that perform prospective audit and feedback, does your ASP team review orders for all antimicrobials or just specific drugs / drug classes (e.g., carbapenems)? *Select one*

[ ] We review all antimicrobial orders [ ] Unknown

[ ] We review antimicrobial orders for specific drugs /or drug classes. *Describe included drugs / classes*

1. Are there any other circumstances besides antimicrobial drug orders that initiate prospective audit and feedback? *Check all that apply*

[ ] Yes, microbiology results (e.g., blood cultures) will also trigger a review

*Please explain*

[ ] Yes, certain lab tests (e.g., procalcitonin) will also trigger a review

*Please explain* [ ] Yes, other *Please explain* [ ] No

1. Over the course of a month, approximately what proportion of all antimicrobial orders does your ASP team review? *Select one*

| [ | ] 1-24% |
| --- | --- |
| [ | ] 25-49% |
| [ | ] 50-74% |
| [ | ] 75-99% |

1. When the ASP team needs to make a recommendation (e.g., intervention), who usually gives the recommendation? *Check all that apply*

[ ] ASP pharmacist [ ] ASP physician

[ ] ASP pharmacist and ASP physician together [ ] Other, pharmacist or physician

[ ] Nurse / Nurse practitioner [ ] Hospitalist

[ ] Other, *describe:*

1. Over the course of a month, approximately what proportion of the ASP team’s recommendations are done using the following approaches? *Put an ‘X’ in the box that corresponds with the proportion. Categories do not need to add up to 100%.*
   1. Email:

[ ] None; [ ] 1-24%; [ ] 25-50%; [ ] 51-74%; [ ] 75-99%; [ ]100%

- 1. Telephone (calling and speaking with the clinician or leaving voice message): [ ] None; [ ] 1-24%; [ ] 25-50%; [ ] 51-74%; [ ] 75-99%; [ ]100%
  2. Text message/ instant message:

[ ] None; [ ] 1-24%; [ ] 25-50%; [ ] 51-74%; [ ] 75-99%; [ ]100%

- 1. EHR alerts or notes:

[ ] None; [ ] 1-24%; [ ] 25-50%; [ ] 51-74%; [ ] 75-99%; [ ]100%

- 1. Face-to-face (handshake stewardship):

[ ] None; [ ] 1-24%; [ ] 25-50%; [ ] 51-74%; [ ] 75-99%; [ ]100%

- 1. Other:

[ ] None; [ ] 1-24%; [ ] 25-50%; [ ] 51-74%; [ ] 75-99%; [ ]100%

## DIAGNOSTIC TESTING / DIAGNOSTIC STEWARDSHIP

1. Does your hospital or health system have any procedures in place to optimize the appropriate use of diagnostic tests? *Select one*

*Note: The procedures may include pre-agreed institutional criteria to limit testing for patients with certain clinical or syndromic criteria*. *The intent is to modify the process of ordering and / or performing diagnostic tests to ensure appropriate testing and improve the care of the patient.*

[ ] No, we do not have any procedures targeting diagnostic testing *Skip to question 38*

[ ] Unknown *Skip to question 38*

[ ] Yes

1. Does your hospital have any procedures in place to prevent inappropriate diagnostic testing for

*Clostridioides difficile* infection?

[ ] No *Skip to question 34*

[ ] Unknown *Skip to question 34*

[ ] Yes

1. Please select the mechanism(s) or strategy(s) your hospital uses to reduce inappropriate diagnostic testing for *Clostridioides difficile*? *Check all that apply*

[ ] Clinician education

[ ] Clinician or nursing checklist to determine appropriateness [ ] Hospital policy created addressing appropriate testing

[ ] Required documentation of clinical factors indicating need for test

[ ] Electronic health record (EHR alert e.g., pop-up) and clinical decision support system (CDSS) [ ] Pre-authorization by ASP or others

[ ] Laboratory-initiated interventions (e.g. refusal to process inappropriately collected or handled specimens, cancelling tests if sample not received within certain time period, cascading)

[ ] Unknown

[ ] Other, *describe:*

1. Does your hospital have procedures to prevent inappropriate diagnostic testing of urine specimens?

[ ] No *Skip to question 36*

[ ] Unknown *Skip to question 36*

[ ] Yes

1. Please select the mechanism(s) or strategy(s) your hospital uses to reduce inappropriate diagnostic testing of urine specimens. *Check all that apply*

[ ] Clinician education

[ ] Clinician checklist to determine appropriateness

[ ] Hospital guideline addressing when urine testing is indicated

[ ] Hospital guideline addressing appropriate methods of urine collection [ ] Required documentation of clinical factors indicating need for test

[ ] Electronic health record alert and / or clinical decision support system (CDSS) [ ] Prescriber attestation that clinical criteria are met

[ ] Antimicrobial stewardship program or Infection Control-led audit and feedback

[ ] Laboratory-initiated interventions (e.g. refusal to process inappropriately collected or handled specimens)

[ ] Removal of specific tests in EHR

[ ] Reflex urine cultures only when specific parameters are met on the urinalysis [ ] Unknown

[ ] Other, *describe:*

1. Does your hospital have procedures to prevent the inappropriate use of molecular diagnostic tests (e.g., respiratory film array panels and meningitis/encephalitis panels)? Please do not consider procedures targeting *Clostridioides difficile* testing.

[ ] No

[ ] Unknown

[ ] Yes, *which tests*?

1. Does your hospital have procedures to prevent the inappropriate use of other diagnostic tests?

[ ] No

[ ] Unknown

[ ] Yes, *which tests?*

**MEASUREMENT OF ANTIMICROBIAL UTILIZATION DATA**

1. Does your ASP team routinely collect any antimicrobial usage data? *Select one*

[ ] No, we do not routinely collect antimicrobial usage data. *Skip to question 48*

[ ] Unknown *Skip to question 48*

[ ] Yes

1. Does your ASP team collect usage data for antimicrobials prescribed in all units (facility-wide) or just specific units / services in the hospital? *Select one*

*Note: For this question, emergency department, pre-op, and post-acute care units are not considered units of the hospital.*

[ ] We collect antimicrobial usage data for all units [ ] Unknown

[ ] We collect antimicrobial usage data for some units / services *Describe which units or services are included and / or excluded*

1. Does your ASP collect usage data for all antimicrobials or for specific drugs / drug classes? *Select one*

[ ] We collect usage data for all antimicrobials [ ] Unknown

[ ] We collect usage data only for specific drugs / drug classes of antimicrobials. *Describe*

_ [ ] Other, *describe*

1. Is your hospital enrolled in the National Healthcare Safety Network (NHSN) optional Antimicrobial Use module through which you can measure the Standardized Antimicrobial Administration Ratio (SAAR)?

[ ] No

[ ] Unknown [ ] Yes

1. Which of the following antimicrobial usage metrics does your facility collect? *Check all that apply*

*Note: Days present is the number of patients who were present for any portion of each day in a specific patient care location. Patient days is the number of patients who were present in a specific patient care location during the once daily census count.*

[ ] Defined daily dose (DDD) per 1000 patient days [ ] Defined daily dose (DDD) per 1000 days present [ ] Days of therapy per 1000 patient days

[ ] Days of therapy per 1000 days present (as required by NHSN)

[ ] Antimicrobial expenditures (e.g., purchasing costs)

[ ] Other, *describe:*

[ ] None

1. What is the source of the numerator used in your usage metric? *(may select more than one answer if you have more than one metric)*

[ ] Pharmacy purchasing records, electronic

[ ] Pharmacy purchasing records, manual review [ ] Electronic aggregated dispensing data

[ ] Manual counting

[ ] EHR Medication Administration Records (MAR)

[ ] Charges for medication billed during acute care stay

[ ] Other, *describe:*

1. What is the source of the denominator for your antimicrobial usage metrics?

[ ] Administrative data from EHR (admission, transfer, discharge data) [ ] Infection control staff

[ ] Manual counting

[ ] Billing or finance data

1. Does your ASP team monitor trends in antimicrobial usage over time (longitudinally)?

[ ] No, ASP team does not monitor trends in antimicrobial usage over time *Skip to question 48*

[ ] Unknown *Skip to question 48*

[ ] Yes, ASP team monitors trends in antimicrobial usage over time

1. Which antimicrobial usage trends do you monitor? *Check all that apply*

[ ] We monitor house-wide trends

[ ] We monitor unit-specific or service-specific trends [ ] We monitor drug or drug class-specific trends

[ ] We monitor provider-specific trends [ ] Unknown

1. How does your ASP team use information related to antimicrobial utilization? *Check all that apply*

[ ] To evaluate the effectiveness of ASP interventions

[ ] To look for overuse or underuse of certain drug classes

[ ] To report to the NHSN (optional Antimicrobial Use module) [ ] Other, *describe:*

__

**MEASUREMENT OF *CLOSTRIDIOIDES DIFFICILE* INFECTION**

1. Does your hospital report hospital-onset *Clostridioides difficile* infection rates to the NHSN? *Select one*

[ ] No, we do not report hospital-onset *Clostridioides difficile* infection rates [ ] Unknown

[ ] Yes, s*kip to question 50*

1. If your hospital does not report *Clostridioides difficile* infection rates to the NHSN, do you measure this rate using another approach?

[ ] No

[ ] Unknown

[ ] Yes, *describe:*

1. How does the ASP team use the information related to *Clostridioides difficile* infection rates? *Check all that apply*

[ ] To evaluate the effectiveness of ASP interventions

[ ] To look for overuse or underuse of certain drug classes

[ ] To look for opportunities to improve *Clostridioides difficile* testing [ ] To report to the NHSN

[ ] Other, *describe:*

1. Optional: Please provide any additional general comments or comments about specific questions in the space below.

Thank you very much for taking the time to provide us with the information requested in this questionnaire. Your participation is a significant contribution to efforts to improve antimicrobial stewardship and patient safety.

**eMethods 2 - Algorithms**

**eMethods 3**

*Questionnaire implementation*

Hospitals were invited to participate in two stages. On 1/29/20, the Joint Commission Department of Research’s senior leadership sent a hard-copy letter to the hospital’s primary accreditation contact (usually director of quality or similar title) introducing the study and stating they would receive an email message requesting participation and the ASP leader’s contact information. The recruitment email invitation on 2/19/20 included confidentiality and voluntary participation statements indicating separation between research and accreditation activities. Thereafter, a 50-item questionnaire was sent via Qualtrics^XM^ platform to the designated ASP leader in hospitals that agreed to participate. Up to four follow-up emails were sent to non-respondents through August 2020.

**eTable 1: Monitoring Adherence for Facility-specific Treatment Guidelines**

| **Condition addressed in facility-specific treatment guideline** | **Developed Guideline**  **No. (%) (n = 288)** | **Monitors Adherence**  **No. (%) (n = 288)** |
| --- | --- | --- |
| Specific condition addressed in guideline: |  |  |
| Community-acquired pneumonia | 246 (85.4) | 70 (24.3) |
| Sepsis | 232 (80.6) | 71 (24.7) |
| Urinary tract infection and / or asymptomatic bacteriuria | 215 (74.7) | 73 (25.3) |
| Surgical prophylaxis | 212 (73.6) | 60 (20.8) |
| Hospital or ventilator-associated pneumonia | 207 (71.9) | 38 (13.2) |
| Skin and soft tissue infection | 199 (69.1) | 46 (16.0) |
| *Clostridioides difficile* infection | 193 (67.0) | 60 (20.8) |
| Intra-abdominal infections (e.g., appendicitis) | 146 (50.7) | 26 (9.0) |
| Febrile neutropenia | 129 (44.8) | 18 (6.3) |
| Meningitis | 125 (43.4) | 19 (6.6) |
| Other | 59 (20.5) | 19 (6.6) |
| At least one condition | 268 (93.1) | 127 (44.1) |
| CAP, UTI, SSTI, and sepsis | 161 (55.9)  (all four) | 107 (37.2)  (at least one of the four) |

Abbreviations: CAP, community-acquired pneumonia; UTI, urinary tract infection and / or asymptomatic bacteriuria; SSTI, skin and soft tissue infection

Percentages are unweighted

| **Mechanisms to optimize diagnostic testing** | **No. (%) (n = 288)** |
| --- | --- |
| Hospital has procedure(s) to prevent inappropriate testing for *Clostridioides difficile* infection | 190 (66.0) |
| Mechanism(s) to prevent inappropriate testing for *C. difficile* infection^a^ | |
| Clinician education | 162 (56.3) |
| Clinician or nursing checklist to determine appropriateness | 117 (40.6) |
| Hospital policy on appropriate testing | 102 (35.4) |
| Required documentation of clinical factors indicating need for test | 80 (27.8) |
| Electronic health record (EHR alert e.g., pop-up) and / or CDSS | 120 (41.7) |
| Pre-authorization by ASP or others | 19 (6.6) |
| Laboratory-initiated interventions^b^ | 165 (57.3) |
| Other | 8 (2.8) |
| Hospital has procedure(s) to prevent inappropriate testing of urine specimens | 112 (38.9) |
| Mechanism(s) to prevent inappropriate testing of urine specimens^a^ | |
| Clinician education | 87 (30.2) |
| Clinician checklist to determine appropriateness | 21 (7.3) |
| Hospital guideline addressing when urine testing is indicated | 45 (15.6) |
| Hospital guideline addressing appropriate methods of urine collection | 32 (11.1) |
| Required documentation of clinical factors indicating need for test | 16, (5.6) |
| Electronic health record alert and / or CDSS | 34 (11.8) |
| Prescriber attestation that clinical criteria are met | 7 (2.4) |
| Antimicrobial stewardship program or infection control-led audit and feedback | 31 (10.8) |
| Laboratory-initiated interventions | 26 (9.0) |
| Removal of specific tests in EHR | 14 (4.9) |
| Reflex urine cultures only when specific parameters are met on the urinalysis | 91 (31.6) |
| Other | 5 (1.7) |
| Unknown | 1 (0.3) |

**eTable 2**: **Mechanisms to Optimize Diagnostic Testing**

Abbreviations: EHR, electronic health records; CDSS, clinical decision support system

Percentages are unweighted

^a^Respondents were asked to select all applicable responses

^b^Laboratory-initiated interventions (e.g., refusal to process inappropriately collected or handled specimens, cancelling tests if sample not received within certain time period, cascading)
